# Ptychographic X-ray nanotomography quantifies mineral distributions in human dentine

**DOI:** 10.1038/srep09210

**Published:** 2015-03-20

**Authors:** I. Zanette, B. Enders, M. Dierolf, P. Thibault, R. Gradl, A. Diaz, M. Guizar-Sicairos, A. Menzel, F. Pfeiffer, P. Zaslansky

**Affiliations:** 1 Physik-Department & Institut für Medizintechnik, Technische Universität München, 85748 Garching, Germany; 2 Diamond Light Source, Harwell Science and Innovation Campus, Didcot, OX11 0DE, United Kingdom; 3 Department of Physics & Astronomy, University College London, WC1E 6BT London, United Kingdom; 4 Paul Scherrer Institut, 5232 Villigen PSI, Switzerland; 5 Institut für diagnostische und interventionelle Radiologie, Klinikum rechts der Isar, Technische Universität München, 81675 München, Germany; 6 Julius Wolff Institute and Center for Musculoskeletal Surgery, Charité - Universitätsmedizin Berlin, 13353 Berlin, Germany

## Abstract

Bones are bio-composites with biologically tunable mechanical properties, where a polymer matrix of nanofibrillar collagen is reinforced by apatite mineral crystals. Some bones, such as antler, form and change rapidly, while other bone tissues, such as human tooth dentine, develop slowly and maintain constant composition and architecture for entire lifetimes. When studying apatite mineral microarchitecture, mineral distributions or mineralization activity of bone-forming cells, representative samples of tissue are best studied at submicrometre resolution while minimizing sample-preparation damage. Here, we demonstrate the power of ptychographic X-ray tomography to map variations in the mineral content distribution in three dimensions and at the nanometre scale. Using this non-destructive method, we observe nanostructures surrounding hollow tracts that exist in human dentine forming dentinal tubules. We reveal unprecedented quantitative details of the ultrastructure clearly revealing the spatially varying mineralization density. Such information is essential for understanding a variety of natural and therapeutic effects for example in bone tissue healing and ageing.

The skeletal elements of all mammals are made of nanocomposites incorporating a carbonated apatite (dahllite) mineral. Typical bone tissues comprise mineralized collagen nanofibrils, where the protein backbone is stiffened and embedded in nanometre sized tablets of dahllite^1^. The degree of mineralization is an important contributor to the mechanical performance of the tissue and has been the subject of investigation for many decades mainly in human bone^2^. Together with fibrillar orientation, the mineral density defines the mechanical properties of bones, including toughness and susceptibility to fracture, hardness and stiffness. In healing bones or osteoporosis for example, the degree of mineralization is a strong indicator of the state of tissue pathology or the progress of biological repair^3^. Consequently, medical treatment is often directed at controlling or increasing tissue mineral content, using measures of mineral density as indicators of therapy needs^4^.

In the crowns of human teeth bone tissue forms the dentine matrix that surrounds the pulp and supports an outer layer of hard and brittle enamel^5^ as can be seen in Fig. 1. The matrix houses parallel, micrometre-thick tubules, extending outwards from the pulp and often confined by a micrometre-thick rim of collagen-free mineral. Dentine is thus a naturally occurring intertubular bony tissue (intertubular dentine, ITD) with regularly spaced high-density mineral columns (peritubular dentine, PTD) surrounding empty (or dental liquid-filled) voids^6^. Much is known about dentine structure and chemistry, and efforts are continuously made to obtain reliable and precise measurements of the mineral characteristics, ideally in three dimensions^7^,^8^.

The most widely used methods to quantify mineral density in bony tissues are quantitative backscattered electron microscopy imaging (BSE)^9^ and X-ray absorption microCT^10^. The former method benefits from the nanometre resolution provided by electron microscopy methods, yet it suffers from being limited to analysis on two dimensional (2D) surface slices, providing no tissue depth information. In addition, BSE requires sufficient tissue volume to backscatter the electrons, typically micrometres in depth, and relies on the use of careful tissue stabilization and sample preparation procedures. Absorption microCT, on the other hand, has the advantage of providing 3D information non-destructively but suffers from poor sensitivity at high resolutions, as the signal collected is inherently weak, even when using high-intensity and monochromatic X-ray sources such as synchrotron facilities.

Penetrating a multi-micrometre thick mineralized sample requires X-rays with a photon energy of at least a few kiloelectron volts. At this energy, the sample refracts the incident X-ray waves much more efficiently than it attenuates them, rendering phase contrast methods much more sensitive than absorption methods^11^. Thus, phase contrast X-ray imaging can be used to combine a high sensitivity comparable to BSE with the 3D nature of X-ray absorption micro computed tomography (microCT).

Here, we explore the potential of ptychographic X-ray nanoCT (PXCT)^12^ to reveal the three-dimensional mass density distribution within mineralized human tooth dentine. Similar to other X-ray phase contrast methods, e.g., Ref. 13, but without any assumption about tissue composition, PXCT gives direct access to the distribution of the electron density^14^ and thus to the mass density within the specimen (see Methods section). PXCT is used to image juxtaposed fully mineralized tissues with different densities, in 3D, at the micrometre and sub-micrometre length scales.

We measured human dentine specimens by scanning thin (30–50 *μ*m) samples using a coherent micrometre-sized X-ray beam, followed by ptychographic and tomographic reconstruction (see Methods section). Figure 2 shows a comparison of typical BSE and PXCT cross-sectional images from similar premolar bulk crown tooth samples. Viewed orthogonal to the tubule axis, in both images, the air-filled lumen of the dentinal tubules appears in black, while the highly mineralized (bright) rings of PTD are seen to be scattered within the ITD matrix. In a typical volume of interest, we observe a range of density values with specific zones exhibiting mass densities exceeding 2.5 g/cm^3^ corresponding to the PTD.

The spatial resolution of 158 nm in our reconstructed volume has been calculated using the Fourier shell correlation method with the half-bit threshold criterion^15^,^16^. This result has been confirmed through analysis of the signal from the smallest features observed in the PXCT images: a lateral tubule “branch”, known to connect neighbouring tubules in teeth^17^. The profile (Fig. 2 (c)) of the selected branch (indicated with arrows in Fig. 2 (b)) has a full width at half maximum of 190 nm (i.e. about 3 pixels of size 65 nm).

The isotropic nature of information provided by PXCT (given a sufficient number of angular projections) ensures that the same spatial resolution is obtained along orthogonal directions, as is shown in Fig. 3 exhibiting some examples of the fine detail of the 3D data. Orthogonal views of the sample are displayed in Fig. 3 (a) and (b) (see also supplementary Movies 1 to 3) while panels (c) to (e) show 3D color renderings of the reconstructed volume (see also supplementary Movie 4). We observe that the thickness of the PTD cuffs varies greatly around each tubule, ranging from 2 *μ*m (see white arrow in Fig. 3 (b)) to below 200 nm. The PTD appears to be thicker where branching is seen (see Movie 3), and some of the fine branches are seen to enter the ITD matrix and connect to neighbouring tubules. In the supplementary Movies 1 to 3 it can also be seen that some of the finer branches of tubules in the ITD regions are surrounded by a mineralized sheath with a density similar to the density of PTD.

Density fluctuations can be observed in the ITD matrix, possibly corresponding to variations in mineral density observed on 2D sections^18^. To better understand the distribution of densities in the tissue, a histogram of the data is shown in Fig. 4. Four distinct peaks, labeled (I) to (IV) can be seen, corresponding to different materials present in the specimen. To precisely quantify the mass density of the main mineralized components in the sample, we have fitted peaks (I), (III) and (IV) with Gaussian functions. The first peak arises from air outside the sample and inside the tubules and has been used to calibrate the density values of our measurements. The width of the air peak (I) is due to noise and measurement errors, which are stronger outside the specimen than inside it. Thus, the full-width at half maximum of the air peak, equal to 0.15 g/cm^3^, provides an estimate of the lowest limit of the density sensitivity of our data. The central small peak marked II (encircled in green) corresponds to a material that is less dense than the ITD, possibly the lamina-limitans soft-tissue, that lines the interior of the tubules^19^. The presence of this layer is especially visible in Fig. 3 (a) and (b), e.g., within the tubule at the bottom of Fig. 3 (a) indicated by the arrow. Its density of approximately 1.35 g/cm^3^ corresponds to the density of typical organic materials such as collagen^20^.

The two other peaks are associated with ITD (III) and PTD (IV). Fits of these peaks yield density values of *ρ*_ITD_ = 2.13 ± 0.09 g/cm^3^ and *ρ*_PTD_ = 2.59 ± 0.08 g/cm^3^ for the ITD and the PTD respectively (mean ± standard deviation). The density of the ITD falls within the range of values previously reported in the literature^21^,^22^. The density of PTD in our specimen is higher than the interval of 2.16–2.28 g/cm^3^ reported from measurements obtained using density fractionation of disaggregated bovine specimens^6^. We cannot rule out the possibility that human PTD may indeed have a higher density than bovine PTD or that, conversely, some ITD or demineralization may affect results of density fractionation PTD measurements. We stress that PXCT requires no chemical sample preparation, circumventing any risks of demineralization. Furthermore, the true 3D nature of our data makes it possible to precisely pick out the tissue type of interest and to quantitatively separate the different mineralization densities at the nanometre scale, with no need for calibration by chemical standards.

With precise estimates of the densities of the main dentine tissue components, it is possible to derive the volume and mass fractions of ITD and PTD (see Methods section). In our air-dried samples, ITD is composed of collagen and mineral. Consequently and based on the mass density measurements known for each component, we find that the ITD mineral volume fraction is 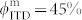 and the mineral mass fraction is 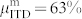, as reported in Table 1. These estimates obtained from the 3D PXCT data are in agreement with the literature, e.g., Refs. 5,7.

For PTD known to include proteolipid-phospholipid complexes^23^ and no collagen, we obtain a mineral volume fraction of 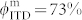 with a mineral mass fraction of 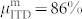, in agreement with results reported in Ref. 23.

From our ultra high resolution, volumetric data by PXCT, it is thus possible to obtain quantitative 3D measurements of mass density in mineralized bony tissues such as those found in human teeth, with an isotropic spatial resolution of less than 200 nm and a density sensitivity of 0.15 g/cm^3^. Such information makes it possible to identify small but important differences in the distribution of mineral within the same specimen and also between different samples. Recent developments in PXCT show that it is possible to achieve resolutions down to 16 nm^24^ thus opening the way to study even finer bone microstructures such as collagen bundles embedded in the mineralized matrix. Our results reveal 20% mineral density differences between PTD and ITD in human dentine, exemplifying the strength of the method to spatially separate juxtaposed differing mineral densities at the micrometre length-scale. PXCT thus combines the advantages of the techniques commonly used to investigate mineralized specimens, i.e. BSE and X-ray absorption microCT, with minimal sample preparation and is likely to forward our understanding of structural variability, bone density variations and bone-tissue mineralization dynamics^25^.

## Methods

### Sample preparation

**Figure 1:**
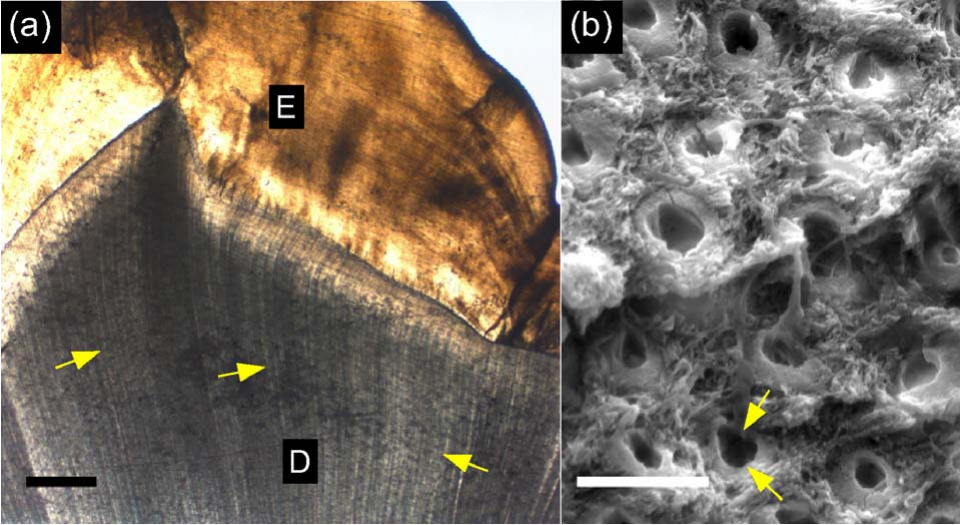
Tubules in bulk dentine. (a) The parallel arrangement of tubules (arrows) in dentine (D) below the enamel (E) is revealed by optical microscopic imaging of a thin polished section. Scale bar: 500 *μ*m (b) The scanning electron microscopy (SEM) image of a fracture surface of dentine reveals tubules (arrows) surrounded by dense peritubular dentine (PTD) with a central empty void. Between tubules, the tissue is occupied by intertubular dentine (ITD) where mineralized collagen fibers are visible. Scale bar: 2 *μ*m.

Human caries-free teeth collected from clinics in accordance with the guidelines provided by the Charité ethical committee from clinics in the greater Berlin area (Germany) were used. Premolars were used for preparation of samples for SEM and PXCT imaging of bulk crown-dentine (circumpulpal), known to have thick PTD. A molar tooth was used to harvest mantle (near-enamel) dentine where PTD is scarce or missing^18^, see Supplementary material. For scanning electron microscopy (SEM), several samples were dehydrated in a series of ethanol solutions, embedded in polymethylmethacrylate, then sliced and polished orthogonal to the tubular orientation. Viewing in a Quanta 600 ESEM (FEI, Eindhoven, The Netherlands) revealed the cross section at a depth of approximately 500 *μ*m below the enamel and dentin-enamel junction. The backscattered SEM image of the uncoated sample (Fig. 1) reveals the typical distribution of tubules and corresponding voids, and highlights subtle variations in mineral content. Sections cut from the buccal (cheek side) of hydrated intact premolar crowns were used for PXCT imaging of bulk crown dentine, based on our previous observations of well developed PTD^18^, while sections cut from buccal cusps of a hydrated intact molar crown were used to prepare samples for imaging mantle dentine. The wet samples were gently manually polished into approximately 50 *μ*m thick plates, from which splinters were cut and mounted upright on holders used for imaging in the beamline.

### Data acquisition

The X-ray ptychography experiments were performed at the cSAXS beamline of the Swiss Light Source, Paul Scherrer Institut, Switzerland. X-rays with an energy of 6.2 keV were used and a 3.2 *μ*m pinhole was placed 4.7 mm upstream of the sample to define a coherent illumination. A PILATUS single-photon counting detector with a pixel side length of 172 *μ*m positioned 7.14 m downstream of the sample recorded diffraction patterns. To reduce X-ray scattering and absorption in air, a tube flushed with Helium was positioned between the sample and the detector.

For each angular view, 565 diffraction patterns were recorded by raster-scanning an area of 62 × 25 *μ*m^2^. A scan with concentric circles was performed to reduce grid artifacts in the reconstructions known to arise due to aliasing by the periodical stepping^26^. The exposure time for each diffraction pattern was 0.1s. This procedure was repeated for 360 evenly spaced angular positions of the sample which rotated over 180°.

### Ptychographic reconstruction

The ptychographic reconstruction was performed with 250 iterations of an algorithm based on the difference map^27^ and a refinement based on the maximum-likelihood approach (250 iterations)^28^. To account for partial coherence in the incident beam, a modal decomposition of the probe into four modes was used^29^. A region of 128 × 128 pixels of the diffraction patterns was used for the reconstruction, resulting in a pixel size in the phase data of 65 nm.

### Tomographic reconstruction

Phase unwrapping, phase ramp removal, and alignment procedures performed on the phase reconstructions prior to tomographic reconstruction are described in Ref. 30. The tomographic reconstructions were performed on differentiated aligned data using the filtered back-projection algorithm with a modified filter^30^.

### From refractive index to mass density and mineral content

The quantity directly measured with PXCT is the decrement of the refractive index *δ*, which, far from the absorption edges of the constituents of the material, is proportional to the material electron density *ρ_e_* = 2*πδ*/(*r_e_λ*^2^) where *ρ_e_* is the classical radius of the electron and *λ* is the wavelength of the radiation (2 Å in our measurements). For bone-like materials^13^, the ratio between molar mass in grams *A* and the atomic number *Z* can be approximated to 2. Thus, the relation between *ρ_e_* and the mass density *ρ* becomes *ρ* = 2*ρ_e_*/*N_A_*, where *N_A_* is the Avogadro constant. In our measurements: 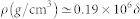.

The mineral content in tooth tissues can be estimated by considering the effects that the constituents of PTD and ITD have on the X-ray beams that we use. For dry bone-like specimens, the signal in the diffraction patterns arises from X-ray interactions with hydroxyapatite crystals and with the organic matter^7^. A variety of density values have been published over more than 150 years for apatite and specifically carbonated apatites (dahllite), and here we use the value of *ρ*_m_ = 3.00 g/cm^3^. Values of density of collagen *ρ*_c_ vary in the interval 1.33–1.45 g/cm^3^, and we consider *ρ*_c_ = 1.40 g/cm^3^ in our analysis. The non-collagenous protein in the PTD, is reported^23^ to have a density of *ρ*_p_ = 1.33 g/cm^3^.

The volume fraction 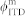 of the mineral in the ITD can be calculated from the relation: 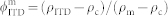. The volume fraction can be further converted into mass fraction using the relation: 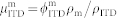.

Analogous relations are used to calculate volume fraction and mass fraction of PTD: 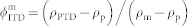 and 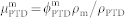.

## Author Contributions

F.P. and P.Z. designed the experiment. I.Z., B.E., M.D., P.T., A.D., M.G.-S. and A.M. performed the experiment. I.Z., B.E., M.D., P.T. and A.D. analysed the data. I.Z., B.E., R.G. and P.Z. wrote the manuscript text. All authors reviewed the manuscript.

## Supplementary Material

Supplementary Information Supplementary Information

Supplementary Information Three-dimensional rendering of dentine sample close to pulp

Supplementary Information Three-dimensional rendering of dentine sample close to enamel

Supplementary Information Axial views through the dentine volume

Supplementary Information Saggital views through the dentine volume

Supplementary Information Coronal views through the dentine volume

## Figures and Tables

**Figure 2:**
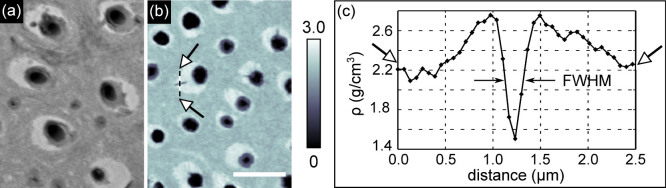
Backscattered electron-microscopy imaging (BSE) versus ptychographic X-ray nanoCT (PXCT) of bulk crown dentine. Highly mineralized islands of peritubular dentine in the intertubular matrix as revealed by (a) BSE microscopy at 10 keV and (b) PXCT at 6.2 keV. The mass density range measured by PXCT is shown in g/cm^3^ in the colorbar of (b). The scale bar is 5 *μ*m for both images. The full width at half maximum (FWHM) of the profile plot taken across one of the branches of the tubules is used in panel (c) to evaluate the spatial resolution as described in the main text.

**Figure 3:**
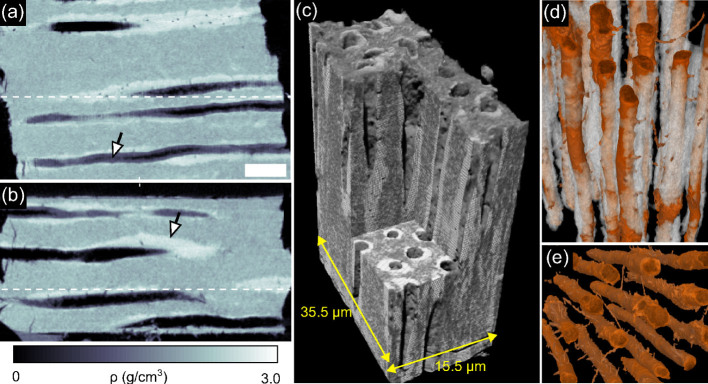
Ptychographic X-ray nanoCT (PXCT) volume rendering of dentine. Panels (a) and (b) show orthogonal views in the PXCT volume. The dashed lines in these panels indicate where these two views intersect. These slices are both orthogonal to the one shown in panel (b) of Fig. 2. The scale bar at the bottom right of panel (a) has a length of 5 *μ*m and a height of 1.5 *μ*m. The arrow in (a) indicates organic debris and the arrow in (b) points to the PTD. The color bar associated to these images is shown below panel (b). Panels (c) to (e) show false-color 3D renderings of the same volume. In particular, (d) and (e) highlight the architecture of the dentinal tubules (with a diameter from 1 to 2 *μ*m) and their branches (diameter of approximately 200 nm).

**Figure 4:**
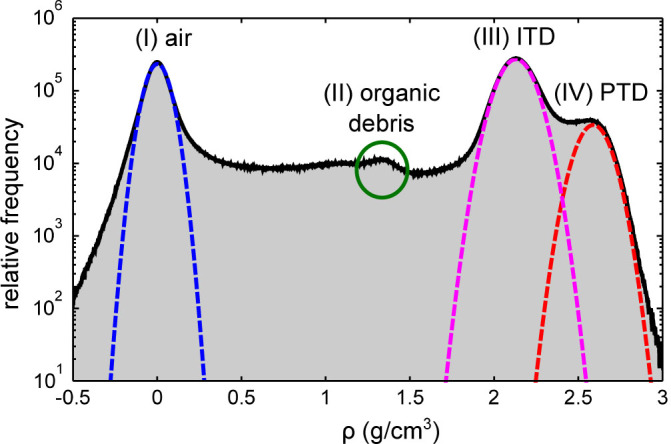
Histogram of dentine obtained by ptychographic X-ray nanoCT. The four peaks (I) to (IV) correspond to air and the three materials typically found in dentine. Gaussian fits to the air (I), intertubular dentine ITD (III) and peritubular dentine PTD (IV) peaks are shown with dashed color lines.

**Table 1:** Results on intertubular (ITD) and peritubular (PTD) dentin. Mass density *ρ*, mineral volume fraction *ϕ*^m^ and mineral mass fraction *μ*^m^ of ITD and PTD calculated from the ptychographic nanoCT volume

	*ρ* (g/cm^3^)	*ϕ*^m^	*μ*^m^
ITD	2.13 ± 0.09	0.45	0.63
PTD	2.59 ± 0.08	0.63	0.86
