# Supplementary material for: Ptychographic X-ray nanotomography quantifies mineral distributions in human dentine
Source: Sci Rep. 2015 Mar 20;5:9210. doi: 10.1038/srep09210 (PMC4366856; doi:10.1038/srep09210)
Supplement: Supplementary Information [file srep09210-s1.pdf]

Ptychographic x-ray nanotomography quantifies mineral distributions in human dentine

I. Zanette[1,2], B. Enders[1], M. Dierolf[1], P. Thibault[1,3], R. Gradl[1], A. Diaz[4], M. Guizar-Sicairos[4], A. Menzel[4], F. Pfeiffer[1,5], and P. Zaslansky[6]

[1] Physik-Department & Institut für Medizintechnik, Technische Universität München, 85748 Garching, Germany
[2] Diamond Light Source, Harwell Science and Innovation Campus, Didcot, OX11 0DE, United Kingdom
[3] Department of Physics & Astronomy, University College London, WC1E 6BT London, United Kingdom
[4] Paul Scherrer Institut, 5232 Villigen PSI, Switzerland
[5] Institut für diagnostische und interventionelle Radiologie, Klinikum Rechts der Isar, Technische Universität München, 81675 München, Germany
[6] Julius Wolff Institute and Center for Musculoskeletal Surgery, Charité - Universitätsmedizin Berlin, 13353 Berlin, Germany

1 Sample preparation

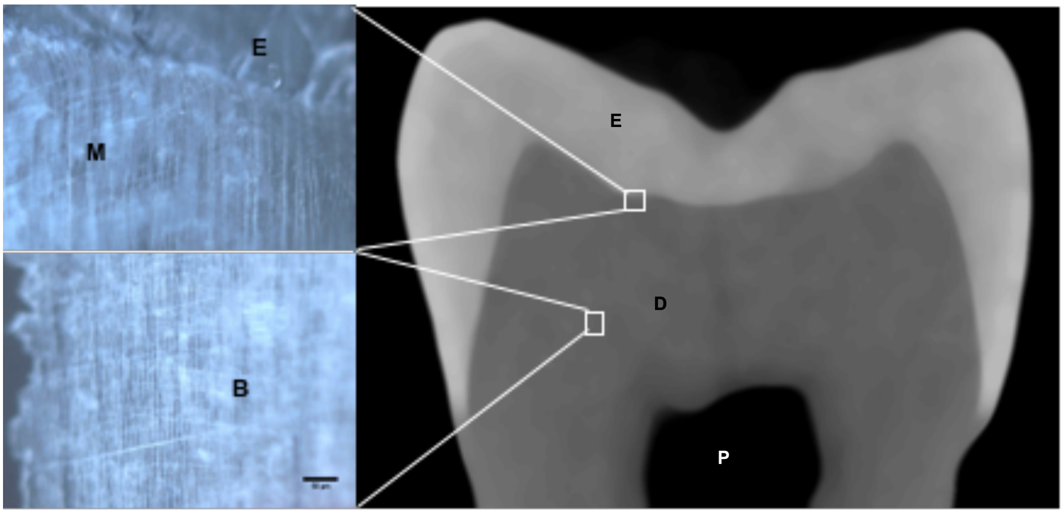

Supplementary Figure 1: **Sample preparation.** Silhouette of a typical human tooth, where dentin (D) is covered by enamel (E) and surrounds the pulp (P). Thin slices were obtained from regions near-enamel containing mantle (M) dentin and from bulk regions (B) containing fully-developed peritubular dentin (PTD). The tubules appear in light microscopy images as thin white lines.

2 Measurements in mantle dentine

A second specimen was measured in this study: a sample of dentine found adjacent to the outer enamel layer and known to have little PTD[1].

The PNCT measurements and reconstructions of this specimen were performed at slightly different parameters than those used for the results reported in the main paper. These differences, which are coincidental, are reported below.

The pinhole had an aperture of 2.2 μm and was situated 3.3 mm upstream of the sample. The PILATUS single-photon counting detector was positioned 7.19 m downstream of the sample.

For each angular view of the sample (180 evenly spaced projections over an angular range of 180 degrees), 612 diffraction pattern collected from an area of $54{\times}24$ μm^2 were recorded each with an exposure time of 0.15 s.

Ptychographic reconstruction was performed with 250 iterations of the

difference map algorithm and 400 iterations of the maximum-likelihood approach. Four probe modes were used in the reconstruction.

Orthogonal slices in the phase volume of the mantle-dentine specimen are shown in Fig. 2. As expected in this specimen, the diameter of the tubules is only about 1.0 μm and thus thinner than in the bulk dentine specimen (Figs. 2 and 3). Also the thickness of the PTD cuffs is much smaller and they are almost absent in this region of the tooth.

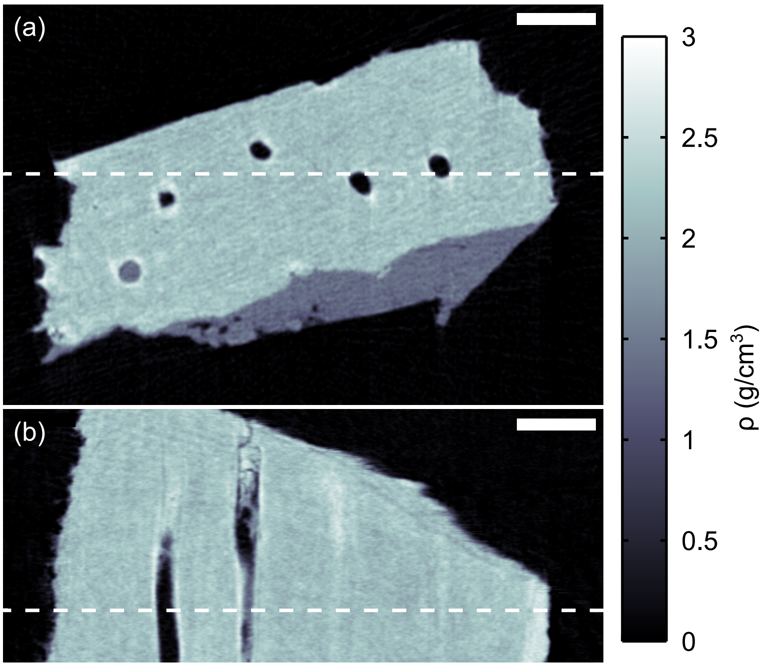

Supplementary Figure 2: **Slices from PNCT measurement of mantle dentine.** Panels (a) and (b) show two representative orthogonal views of the volume measured with PNCT. The scale bars are 5 μm in length.

The histogram of the density phases found in mantle dentine (Fig. 3) shows the same peaks as the histogram on the bulk dentine (see main text), i.e. an air peak (I), a low-density peak corresponding to non-mineralized material (II), the ITD (III) and PTD (IV) peaks. The histogram of bulk dentine is plotted as an overlay, for ease of comparison (gray line on the same graph). Similar to bulk dentine, the air peak in the tubules in mantle dentine serves for calibration, its FWHM of 0.14 g/cm^3 is comparable to that of our other measurements. The result of the fit of peak (II) confirms that the density of the organic material (in this sample, some additional low-density debris, is seen to surround the sample, see Fig. 2) is of 1.33$\pm$0.07 g/cm^3. The ITD has an average density of 2.17$\pm$0.10 g/cm^3, slightly but insignificantly

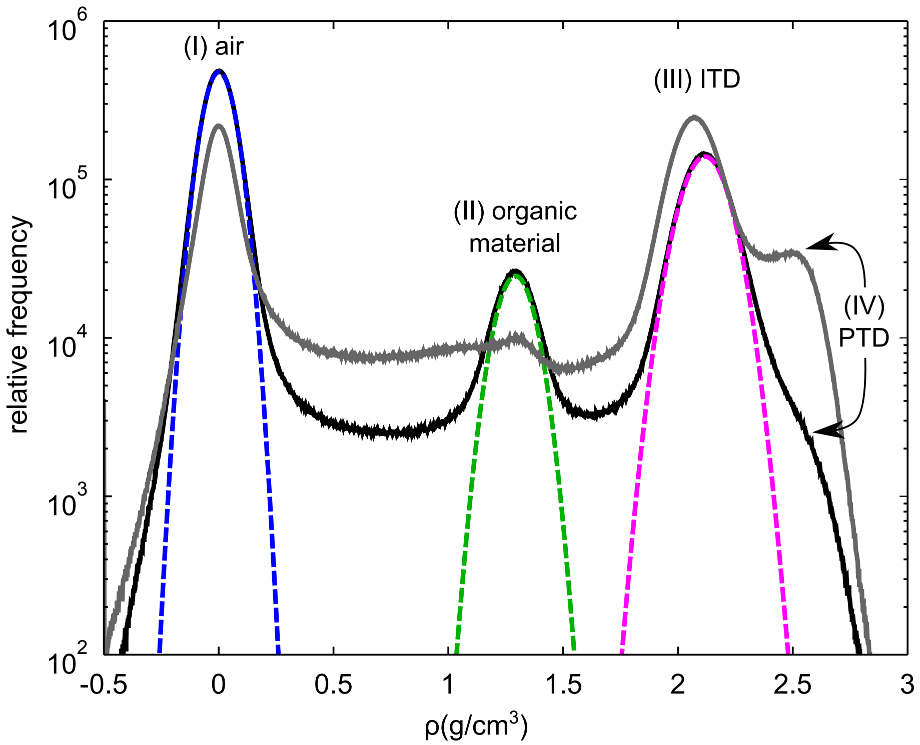

Supplementary Figure 3: **Histogram of mantle dentine.** The black line shows the histogram of the representative mantle dentine volume. The fits of the air, the organic material, and ITD peaks are shown with dashed lines. The grey curve displays the histogram of the volume of the bulk crown dentine sample discussed in the main paper.

higher than the density of the ITD that we found in bulk dentine.

Because the PTD layer is much thinner for mantle dentine than for bulk dentine, the PTD peak is much lower. From comparison with the grey plot of the two histograms, however, we note that the PTD density in mantle dentine appears to be comparable to the PTD density in bulk dentine.

References

1. Weber, D. F. The distribution of peritubular matrix in human coronal dentin. *J. Morph.* **126**, 435-445 (1968).
